# Pemphigus: Current and Future Therapeutic Strategies

**DOI:** 10.3389/fimmu.2019.01418

**Published:** 2019-06-25

**Authors:** Dario Didona, Roberto Maglie, Rüdiger Eming, Michael Hertl

**Affiliations:** ^1^Department of Dermatology and Allergology, Philipps University, Marburg, Germany; ^2^Surgery and Translational Medicine, Section of Dermatology, University of Florence, Florence, Italy; ^3^Section of Dermatology, Departement of Health Sciences, University of Florence, Florence, Italy

**Keywords:** pemphigus, CAAR T-cell, rituximab, anti-CD 20 antibodies, BTK inhibitors, neonatal Fc receptor (FcRn)

## Abstract

Pemphigus encompasses a heterogeneous group of autoimmune blistering diseases, which affect both mucous membranes and the skin. The disease usually runs a chronic-relapsing course, with a potentially devastating impact on the patients' quality of life. Pemphigus pathogenesis is related to IgG autoantibodies targeting various adhesion molecules in the epidermis, including desmoglein (Dsg) 1 and 3, major components of desmosomes. The pathogenic relevance of such autoantibodies has been largely demonstrated experimentally. IgG autoantibody binding to Dsg results in loss of epidermal keratinocyte adhesion, a phenomenon referred to as acantholysis. This in turn causes intra-epidermal blistering and the clinical appearance of flaccid blisters and erosions at involved sites. Since the advent of glucocorticoids, the overall prognosis of pemphigus has largely improved. However, mortality persists elevated, since long-term use of high dose corticosteroids and adjuvant steroid-sparing immunosuppressants portend a high risk of serious adverse events, especially infections. Recently, rituximab, a chimeric anti CD20 monoclonal antibody which induces B-cell depletion, has been shown to improve patients' survival, as early rituximab use results in higher disease remission rates, long term clinical response and faster prednisone tapering compared to conventional immunosuppressive therapies, leading to its approval as a first line therapy in pemphigus. Other anti B-cell therapies targeting B-cell receptor or downstream molecules are currently tried in clinical studies. More intriguingly, a preliminary study in a preclinical mouse model of pemphigus has shown promise regarding future therapeutic application of Chimeric Autoantibody Receptor T-cells engineered using Dsg domains to selectively target autoreactive B-cells. Conversely, previous studies from our group have demonstrated that B-cell depletion in pemphigus resulted in secondary impairment of T-cell function; this may account for the observed long-term remission following B-cell recovery in rituximab treated patients. Likewise, our data support the critical role of Dsg-specific T-cell clones in orchestrating the inflammatory response and B-cell activation in pemphigus. Monitoring autoreactive T-cells in patients may indeed provide further information on the role of these cells, and would be the starting point for designating therapies aimed at restoring the lost immune tolerance against Dsg. The present review focuses on current advances, unmet challenges and future perspectives of pemphigus management.

## Introduction

### Definition

Pemphigus encompasses a heterogeneous group of autoimmune chronic blistering skin diseases, which affect both mucous membranes and the skin. Pemphigus group diseases are characterized by IgG autoantibodies directed against epidermal adhesion complexes (desmosomes) of keratinocytes, leading to loss of cell–cell adhesion, a phenomenon called acantholysis (1, 2).

Pemphigus can be divided into three major forms: pemphigus vulgaris (PV), pemphigus foliaceus (PF), and paraneoplastic pemphigus (PNP). Autoantibodies directed against Dsg3 and Dsg1 are mainly identified in PV; anti-Dsg1 autoantibodies are the serological hallmark of PF (3). In addition, autoantibodies targeting non-Dsg antigens have been reported in PV patients (4), such as IgG against alpha9 acetylcholine receptor (5), various mitochondrial nicotinic cholinergic receptor subtypes (4) and desmocollins 1-3 (4).

A variety of IgG autoantibodies have been described in PNP patients, including IgG against adhesion proteins of the plakin family, plakophilin 3, desmocollins 1 and 3, Dsg1, and Dsg3 and a 170 kD protein which has been recently identified as the protease inhibitor, alpha-2 macroglobulin-like 1 (A2ML1) (6).

Alike PV, PF and PNP, IgA pemphigus is an extremely rare variant of pemphigus, in which IgA but not IgG autoantibodies against epidermal antigens can be identified (3, 7, 8).

### Epidemiology

#### Epidemiology of Pemphigus Vulgaris

PV is the most common clinical pemphigus variant. The annual incidence rate has been reported between 0.76 and 16.1 per million population, depending on the geographical area and the ethnicity (9, 10), with the highest incidence reported in Ashkenazi Jews (10, 11). This observation has been related to the more frequent occurrence of particular Human Leukocyte Antigen (HLA) class II genes in PV patients of Jewish origin, particularly HLA-DRB1^*^04:02; while HLA-DQB1^*^05:03 is more common in non-Jewish PV patients (12) and was also shown to have the strongest association with PV in a Chinese study using next generation sequence analysis (13).

The exact prevalence of pemphigus is unknown. A German analysis reported a point prevalence of 0.009% (14), while a Danish analysis estimated the pemphigus prevalence at 0.006% (15, 16). In addition, in a recent analysis on the US population, an overall standardized point prevalence of 5.2 cases per 100,000 adults has been reported (17). The age at initial PV presentation varies from 36.5 and 72.4 years (12). The mean age of PV onset is 50–60 years, although several cases of PV in children have been described (12). A female predominance has been globally reported, with an estimated female to male ratio of 5.0 in the American PV population (11).

#### Epidemiology of Pemphigus Foliacues

The annual incidence of sporadic PF in the Caucasian population is ~0.04 per 100.000 inhabitants (10, 12). Sporadic PF corresponding to ~20% of pemphigus cases (10, 12). People in the fifth decade are mainly affected, without sex preference (10, 12). HLA-DRB1^*^04:01, HLA-DRB1^*^04:06, HLA-DRB1^*^14, DRB1^*^01:01, have been associated with a higher risk of PF (13, 18). No ethnic predisposition has been reported (10, 12).

Endemic PF (*fogo selvagem*) has been reported in some areas of Brazil, Colombia, and Tunisia (19). Most of the patients are young rural workers, who live in forest areas adjacent to rivers and streams (19). In these areas, some insects including black fly (simulium species), are though to trigger the disease, leading to an immune reaction against Dsg1 via molecular mimicry (20, 21). This hypothesis is supported by high positivity rates of anti-Dsg1 IgG autoantibodies in the sera of healthy individuals living in endemic regions of *fogo selvagem* (21). In Brazilian population HLA-DRB1 alleles ^*^04:04, ^*^14:02, ^*^14:06, and ^*^01:02 have been reported as risk factors for *fogo selvagem* (22).

#### Epidemiology of Paraneoplastic Pemphigus

PNP is considered a rare disease, with about 500 cases reported in the literature (6, 23). Patients between 45 and 70 years of age are usually affected (6, 23). PNP accounts for 3–5% of all pemphigus cases (6, 23). Furthermore, PNP can affect also children and adolescents, particularly in association with Castleman's disease (6, 23). In this sub-group of patients, a predisposition in patients with Hispanic roots was described (24). An association with HLA class II DRB1^*^03 and HLA Cw^*^14, respectively, was reported in Caucasian and in Han Chinese patients (25, 26).

### Major Clinical Variants

#### Pemphigus Vulgaris

**Figure 1:**
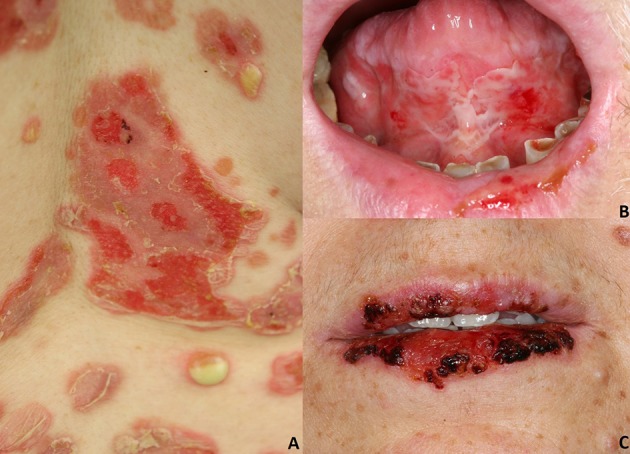
Pemphigus vulgaris: **(A)** Flaccid cutaneous blisters associated with erosions; **(B)** Multiple erosions of the tongue and of the lips; Paraneoplastic pemphigus: **(C)** haemorrhagic crusts and erosion of the lips. All the patients gave written informed consent for the publication of the pictures.

More than half of the patients develop flaccid cutaneous blisters (3, 8, 27) (Figure 1A), which evolve into oozing erosions on erythematous skin. The entire skin may be affected, although lesions mostly occurs in areas exposed to increased mechanical stress (e.g., intertriginous areas) (3, 8, 27) and seborrheic areas (3, 8, 27). Bacterial or viral superinfections of cutaneous and mucosal lesions are fairly common. Cutaneous blisters and erosions usually transform into crusts followed by re-epithelisation without scars. Post-inflammatory hypo and/or hyperpigmentation are common.

In most instances, PV initially manifests with extremely painful erosions of the oral mucosa, particularly the buccal mucosa, the gingiva, the tongue, and the hard and soft palate (3, 8, 27) (Figure 1B). These lesions lead impaired food uptake which results in progressive weight loss. Hoarseness of the voice may be indicative of laryngeal involvement. In the early stages, oral lesions may be misinterpreted as recurrent aphthae, herpetic gingivostomatitis, or erosive lichen planus (3, 8, 27). Other mucous membranes might be less frequently involved, such as laryngeal, esophageal, conjunctival, nasal, anal, and genital mucosa (28).

PV may also involve the nail apparatus. In one study, nail involvement occurred in circa 13% of PV patients. Nail alterations included paronychia, nail discoloration, onychorrhexis, periungual hemorrhages, and onycholysis (29).

**Figure 2:**
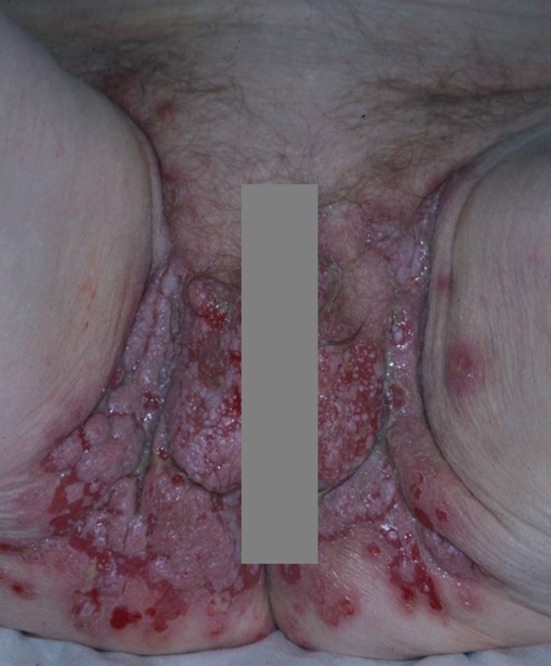
Pemphigus vegetans: vegetative lesions and erosions of the groin and genitals.

Erosions of the intertriginous areas, the scalp and face might evolve into papillomatous or vegetative lesions characterized by abnormal growth of keratinocytes (30) (Figure 2). This phenomenon represents the clinical hallmark of pemphigus vegetans (PVe) (30), which accounts for <5% of pemphigus cases (30).

A substantial number of PV patients shows a transition from a mucosal dominant to a mucocutaneous phenotype with skin lesions characteristic of PF as a result of epitope spreading, a process of diversification of B and/or T-cell responses from the initial dominant epitope (i.e., Dsg3) to a secondary one (i.e., Dsg1) (31). Based on the involved area, PV can be clinically divided in mucosal dominant, mucocutaneous, and, less frequently, cutaneous dominant (3, 8, 27).

#### Pemphigus Foliaceus

**Figure 3:**
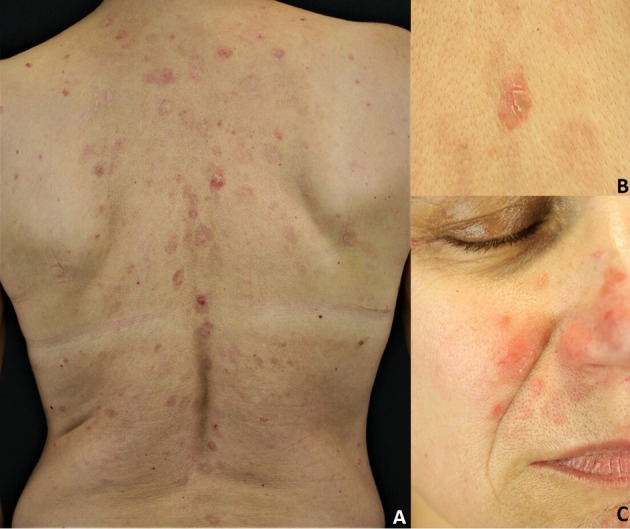
Pemphigus foliaceus: **(A)** Scaly and crusted erythematous plaques on the seborrheic areas; **(B)** Leafy and crusted circumscribed erosion on the back; **(C)** Scaly erythematous plaques on the seborrheic areas. All the patients gave written informed consent for the publication of the pictures.

Sporadic PF is characterized by the absence of mucosal involvement (3, 8, 27). It presents with leafy, scaly and crusted circumscribed erosions on erythematous skin (3, 8, 27) (Figure 3). Seborrheic areas, including the upper trunk and the face, are mainly involved. Flaccid, fragile blisters are rarely seen because of their fragility. Skin lesions can dramatically progress leading to exfoliative erythroderma. PF onset is often subtle, with a few scattered crusted lesions that resembling impetigo. Furthermore, the scaly erythema on the scalp may be misdiagnosed for seborrheic dermatitis. The endemic variant of PF *(fogo selvagem)* is clinically and pathologically indistinguishable from the sporadic one (3, 8, 27). Pemphigus erythematosus (Senear-Usher syndrome) is a rare clinical variant of PF (3, 8, 27), characterized by malar erythemato-squamous plaques and vesicles involving the face in a butterfly-like distribution pattern, the trunk and sun-exposed areas resembling lupus erythematosus (32). In addition, a diagnosis of psoriasis should be also ruled out. Pemphigus seborrhoicus is a very superficial variant of PF with extensive superficial, crusty erosions and erythematous plaques affecting seborrheic areas, particularly the face (3, 27).

#### Paraneoplastic Pemphigus

PNP is a rare pemphigus variant which is always associated with underlying neoplasms, both malignant and benign. Up to 84% of all PNP cases are secondary to hematologic malignancies (33), including non-Hodgkin lymphomas (38.6%), chronic lymphocytic leukemia (18.4%), Castleman's disease (18.4%), thymoma (5.5%), Waldenstrom's macroglobulinemia (1.2%), Hodgkin lymphoma (0.6%), and monoclonal gammopathy (0.6%) (6, 33). Less frequently, epithelial carcinomas (8.6%), sarcomas (6.2%) and gastric cancers have been described in association with PNP (6, 33–35). Some cases of PNP have been reported to be triggered by anti-neoplastic drugs, including fludarabin and bendamustine (36) or radiotherapy (37). PNP has a polymorphic clinical appearance, probably related to the variable presence of different IgG autoantibodies in addition to anti Dsg3/Dsg1 IgG (38). PNP typically presents with a painful stomatitis, and with extensive erosions of the oral cavity and oropharynx (Figure 1C). Usually, the vermillion border of the lips is involved (6). Differential diagnosis includes erythema multiforme (EM), toxic epidermal necrolysis (TEN) and Stevens-Johnson's syndrome; in pediatric cases, oral involvement may be mistaken for a herpetic stomatitis (6).

The nasopharynx, anogenital region, and esophagus may be also affected. Ocular involvement occurs in about 70% of cases (39). Usually, skin lesions, including diffuse erythema, vesicles, blisters, papules, scaly plaques, exfoliative erythroderma, erosions or ulcerations, appear after the onset of the mucosal lesions (38). Moreover, erythema may appear as macular, urticarial, targetoid or polymorphous lesions and a single patient may present different types of lesions, that could evolve from one type to another (40). Lichenoid lesions are also common and occur more frequently in children (24).

The peculiar clinical features of PNP can be explained by both, antibody-driven and cell-mediated pathogenetic mechanisms (41). The first usually determine a PV-like clinical phenotype, while the second features lead to a lichenoid phenotype. More than 90% of PNP cases show an involvement of the respiratory epithelium with dyspnea, obstructive lung disease, and bronchiolitis obliterans, which is one of the main causes of death in PNP (6, 42, 43). Recently, a correlation between bronchiolitis obliterans and anti-epiplakin Ig Abs was found in Japanese PNP patients (42).

#### IgA Pemphigus

IgA pemphigus is characterized by intraepidermal pustules or vesicles with neutrophilic infiltration (3, 7, 8). Acantholysis is usually absent. Depending on the level of pustule formation, IgA pemphigus is divided into two major subtypes, namely subcorneal pustular dermatosis type (IgA-SPD), characterized by subcorneal pustules in the upper epidermis, and intraepidermal neutrophilic type (IgA-IEN), characterized by suprabasilar pustules located at the lower or entire epidermis (3, 7, 8).

### Pathogenesis

#### Dsg1/Dsg3 Compensation Theory

Because of the different expression of the pemphigus autoantigens (Dsg1 and Dsg3) in the cornified and non-cornified epithelium, skin and mucosae are differentially affected by anti-Dsg IgG autoantibodies. PF patients show only anti-Dsg1 IgG autoantibodies; whilst, patients affected by mucosal-dominant PV have only anti-Dsg3 IgG autoantibodies. Furthermore, in patients with mucocutaneous PV both anti-Dsg3 and anti-Dsg1 IgG autoantibodies are detected (44). In the skin and mucosae, the expression of Dsg1 and Dsg3 is different: cutaneous Dsg1 is expressed in the entire epidermis, but more strongly in the superficial layers; cutaneous Dsg3 is expressed in the lower epidermis, mainly in the basal and parabasal layers. On the contrary, mucosal Dsg1 and Dsg3 are expressed in the entire squamous layer, but the expression of Dsg3 is much higher.

Therefore, sera with only anti-Dsg1 IgG lead to superficial blisters and only in the skin, as in PF, because Dsg3 compensates for the loss of Dsg1. In contrast, anti-Dsg3 IgG lead to impairment of mucosal epidermal adhesion because of the low expression of Dsg1, that is not adequate to compensate the loss of Dsg3 adhesion. When anti-Dsg1 and anti-Dsg3 IgG is present, skin and mucous membranes are affected (45).

#### Blister Formation and Acantholysis: Auto-Antibody Dependent Factors

Ig autoantibodies directed against Dsg antigens lead to epithelial acantholysis presumably through several synergistic mechanisms. A model in which acantholysis is produced by interference through antibodies in desmosome adhesion and/or assembly has been proposed. Furthermore, an altered outside-in-signaling caused by antibodies has been thought to cooperate in damaging the desmosomal integrity (46).

The pivotal role of antibodies in pemphigus has been extensively reported (47). Furthermore, it has been highlighted that the sole monovalent antibody fragments can lead to skin lesions (48). In addition, IgG4 antibodies have been mainly reported in pemphigus, which do not involve the complement cascade (49).

The most important targets for Ig antibodies in pemphigus are extracellular domains of Dsg. Dsg show five extracellular cadherin repeats domains (EC1-EC5); the amino-terminal EC1 and EC2 domains, which play a pivotal role in adhesive interactions, are usually targeted by pemphigus antibodies. Indeed, anti-Dsg3 autoantibodies form PV patients and model mice bind directly to residues involved in trans-adhesion (50) and cis-adhesion (51). Thus, antibodies to the NH2-terminal cadherin domains likely compete with or block cellular cohesion. Di Zenzo et al. (51) propose that human anti-Dsg3 autoantibodies bind to the cis-adhesive Dsg3 interface inducing acantholysis. Furthermore, in contrast to IgG autoantibodies directed against other epitopes of Dsg1 and Dsg3, the serum concentrations of these IgG Abs correlate with disease activity (52, 53).

Depletion of Dsg results from several steps: desmosomes lose adhesive properties, probably through a direct interference of trans-interaction of Dsg; further, different signaling pathways cause Dsg endocytosis and depletion, leading to loss of demosomal integrity and adhesion (45). Moreover, the depletion of extradesmosomal Dsg located in association with lipid raft components may affect the *ex novo* expression of desmosomes (54). Furthermore, it has been reported that polyclonal IgG antibodies from PV patients can directly inhibit homophilic Dsg3 trans-interactions. These evidences provide support for the steric hindrance model of pemphigus pathogenesis (52, 53).

Further mechanisms have been thought to be involved in pemphigus acantholysis. Dsg endocytosis and desmosome disassembly have been reported as triggered by both IgG autoantibodies from PV patients and recombinant monovalent human anti-Dsg3 autoantibodies (55, 56). In addition, intercellular widening at non-acantholytic cell layers induced by pathogenic pemphigus antibodies have been detected by both immunofluorescence and electron microscopy findings (57, 58).

Autoantibody-triggered cellular signaling pathways have been also reported as pathogenetic co-mechanisms in pemphigus. Specifically, it has been shown that polyclonal IgG antibodies from serum of PF patients can lead to dissociation of Dsg1 junctions without blocking homophilic Dsg1 trans-interactions (59). In addition, several molecules and signaling pathways have been reported as playing a role in pemphigus acantholysis, including p38 mitogen-activated protein kinase (MAPK) and MAPK-activated protein kinase 2 (MK2) (60, 61). p38MAPK directly modulates intermediate filament formation and the maintenance of the desmosomal structure. It was recently reported that p38 MAPK signaling and Dsg3 internalization play a pivotal role in pemphigus acantholysis (62); however, it has been also highlighted that blisters induced by monoclonal autoantibodies from PV patients are not affected by p38 or MK2 inhibition, indicating that this mechanism of blisters formation might be mainly related to steric hindrance (60, 61, 63).

Furthermore, Saito et al. (63) demonstrated that monoclonal and polyclonal autoantibodies are both complementary involved in acantholysis; indeed, an inducible Dsg clustering has been reported with polyclonal serum IgG, but not with monoclonal antibodies. Furthermore, several other signaling molecules and pathways have been reported as altered by anti-Dsg autoantibodies in pemphigus acantholysis, such as EGFR, caspases (64), and MYC (65). However, none of these events can solely induce acantholysis.

#### Blister Formation and Acantholysis: IgG-Independent Factors

Beside of the Abs role in acantholysis, several autoantibody-independent factors have been thought to be involved in acantholysis. However, the distinct role of these factors is not completely understood.

An increment of Th2 cytokines, such as interleukin (IL)-4, IL-6, and IL-10, has been extensively reported in sera of PV patients, while a reduction of Th1 cytokines, such as IL-2 and Interferon-gamma (IFN-γ) has been also reported (66). In addition, an increase of IL-17a, produced by Th17-cells, and IL-21 and IL-27, synthesized by T follicular helper cell, has been detected (67). Of note, IL-17a was also shown in PV blisters (67). Furthermore, complement activation, cytotoxic proteases and high levels of IL-4 and IL-10 were observed (66, 68). In addition, Tumor Necrosis Factor-alpha (TNF-α) RNA is widely expressed in PV skin lesions and TNF-α serum concentrations correlate with disease activity and IgG autoantibody titers (69, 70).

The importance of apoptosis of epidermal keratinocytes in acantholysis is still under debate. Indeed, some groups considered this process a downstream event after loss of cell–cell adhesion (71), while others suggested it as an upstream event (72). Caspase 8 activation induced by Fas ligand (FasL) detected in PV sera was described to induce apoptosis in keratinocytes. It has been reported *in vitro* and *in vivo* that hindrance of FasL protein causes an inhibition of PV IgG-induced apoptosis of epidermal keratinocytes, suggesting a pivotal role of that FasL in PV pathogenesis (73, 74). Moreover, Lotti et al. (73) highlighted that apoptosis precedes acantholysis, as Fas overexpression, caspase activation before cell detachment *in vivo*.

Furthermore, it was reported that the secretion of cytokines from keratinocytes could be stimulated by PV-IgG (75). Indeed, the expression of the transcription factor ST18 in keratinocytes was reported in response to PV-IgG, leading to both secretion of cytokines and loss of keratinocyte cohesion; therefore, it has been concluded that that cytokines contribute to blistering downstream of autoantibodies (75).

### Diagnostics

The proper diagnosis of pemphigus is based on four criteria, namely clinics, histopathology of the lesional skin, direct immunofluorescence microscopy (DIF) of perilesional skin, and detection of serum autoantibodies by indirect immunofluorescence microscopy (IIF), enzyme-linked immunosorbent assay (ELISA) and/or additional techniques such as Biochip, immunoblot analysis or immunoprecipitation (7, 76) (Table 1).

**Table 1:** Diagnostic algorithm in pemphigus [adapted from Witte et al. (76)].

**HISTOPATHOLOGY**
Suprabasal acantholysis (IgA-IEN, PNP, PV) Subcorneal acantholysis (IgA-SPD, PF) Interface dermatitis with vacuolization of the basal cells and lichenoid infiltrate at the DEJ (PNP)
**DIF**
Reticular binding of IgG and/or C3 to the surface of epidermal keratinocytes (PF, PV) Reticular binding of IgA and/or C3 to the surface of epidermal keratinocytes (IgA-IEN, IgA-SPD) Linear deposits of IgG and/or C3 at the BMZ (PNP) Net-like IgG and/or C3 deposits on the surface of epidermal keratinocytes and along the DEJ (PNP)
**IIF**
Reticular pattern of cell surface reactivity of IgG antibodies on the epithelium of monkey esophagus (PF, PNP, PV) Reticular pattern of cell surface reactivity of IgA antibodies on the epithelium of monkey esophagus (IgA-IEN, IgA-SPD) Net-like pattern of cell surface reactivity of IgG antibodies on monkey esophagus epithelia, normal human skin, and plakin-rich urinary bladder (PNP)
**ELISA**
Alpha-2-macroglobulin-like-1 (PNP) BP230 (PNP) Desmocollin 1 (IgA-SPD) Desmocollin 3 (IgA-SPD, PNP) Desmoglein 1 (IgA-IEN, PF, PNP, PV) Desmoglein 3 (IgA-IEN, PV, PNP) Periplakin/Envoplakin (PNP)

*BMZ, Basal membrane zone; DEJ, dermo-epidermal junction; DIF, direct immunofluorescence; ELISA, enzyme-linked immunosorbent assay; IgA-IEN, intraepidermal neutrophilic type of IgA pemphigus; IgA-SPD, subcorneal pustular dermatosis type of IgA pemphigus; IIF, indirect immunofluorescence; PF, pemphigus foliaceus; PNP, paraneoplastic pemphigus; PV, pemphigus vulgaris*.

**Figure 4:**
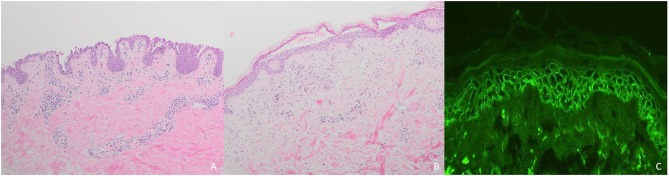
Diagnostic of pemphigus: **(A)** Intraepidermal acantholysis in pemphigus vulgaris; **(B)** Subcorneal loss of adhesion in pemphigus foliaceus; **(C)** Reticular binding of IgG in pemphigus vulgaris.

Histopathologically, PV is characterized by intraepidermal acantholysis (7) (Figure 4A), with basal keratinocytes still attached to the basement membrane zone assuming a characteristic tombstone-like morphology. In contrast to PV, PF lesions show a more superficial, subcorneal acantholysis (Figure 4B). In PNP, the histopathological features are polymorphic. Bullous lesions show suprabasal acantholysis with dyskeratosis and a scattered inflammatory infiltrate (6). In maculopapular lesions, a lichenoid interface dermatitis is more frequently observed (6). Clinically mixed maculopapular and bullous lesions show both acantholysis and lichenoid interface dermatitis (23). IgA pemphigus is characterized by intraepidermal pustules or vesicles with neutrophilic infiltration whereas acantholysis is usually absent (7).

In all pemphigus variants, DIF of perilesional skin shows reticular binding of IgG and/or C3 to the surface of epidermal keratinocytes (7, 77) (Figure 4C). In IgA pemphigus, DIF detects IgA instead of IgG (7, 77). In PNP, net-like IgG and/or C3 deposits on the surface of epidermal keratinocytes and along the dermo-epidermal junction can be detected in <50% of cases (6). In contrast to PV and PF, PNP lesions show linear deposits of IgG and/or C3 at the basal membrane zone by DIF (6).

In IIF routine diagnostics, monkey esophagus is used as the major tissue substrate. A reticular pattern of cell surface reactivity of IgG antibodies with epithelial cells is characteristic (7, 77). In IgA pemphigus, intercellular deposits of IgA directed against Dsg 1 and Dsg 3 (IgA-IEN), as well as against Dsc 1 and Dsc 3 (IgA-SPD) are detected by IIF (7, 77). IgA autoantibodies in IgA-SPD may be detected by IIF on Dsc1-transfected COS-7 cells (78).

In PNP, IgG antibodies directed against plakins can be detected; among those, IgG against envoplakin and periplakin are the most common (23). In PNP, IIF also shows a net-like staining pattern with normal human skin and plakin-rich urinary bladder, the latter being the substrate of choice since it shows for the detection of plakin-reactive IgG autoantibodies (83%) (6, 23, 76).

In PV, IgG autoantibodies against Dsg 1 and Dsg 3 can be detected by ELISA. Patients affected by dominant cutaneous PV show only or preferentially anti-Dsg 1 autoantibodies, while patients with mucosal dominant PV show only or preferably anti-Dsg 3 IgG autoantibodies. In muco-cutaneous PV, both anti-Dsg 1 and anti-Dsg 3 autoantibodies can be detected. In contrast to PV, patients with PF show only IgG against Dsg 1 in the vast majority of cases (7, 77). In general, anti-Dsg 1 and anti-Dsg 3 serum antibody concentrations correlate with disease activity (7, 77). However, in case of atypical pemphigus, autoantibodies against Dscs are detected, while reactivity against Dsg 3 and/or Dsg 1 lacks. In atypical pemphigus, both IgA and IgG against different Dscs are detected (79). However, routine evaluation of serum IgG and IgA against Dscs does not play a significant role in making the diagnosis of PV and PF (79).

Depending on the subtype, different IgA autoantibodies are detected in IgA pemphigus by ELISA, including IgA against Dsc 1, Dsg 1, and Dsg 3.

The spectrum of IgG autoantibodies is more diverse in PNP, including IgG autoantibodies against Dsg 1, Dsg 3, desmoplakin 1, desmoplakin 2, Dsc 1, Dsc 3, envoplakin, periplakin, plectin, BP180, BP230, and the protease inhibitor, alpha-2-macroglobulin-like-1 (6, 80). However, ELISA lacks sensitivity in PNP patients due to the wide range of autoantigens targeted by Ig autoantibodies (81).

Immunoblotting and immunoprecipitation are considered useful techniques for diagnosing PNP and can show IgG antibodies against several antigens, including plakins, periplakin, desmoplakin, BP180, BP230, and alpha-2-macroglobulin-like-1 (6, 80). Indeed, IgG autoantibodies against envoplakin and periplakin and/or alpha-2 macroglobulin-like-1 confirm the diagnosis of PNP. Therefore, in PNP patients two of three serological techniques (IIF on rat bladder, immunoblot and immunoprecipitation) should be performed to establish the correct diagnosis (76).

Immunoblot analysis is performed with recombinant proteins or extracts of dermis, epidermis, bovine gingiva, amnion membrane or cultured keratinocytes (76). They can be used for the detection of several autoantibodies, such as anti-envoplakin, anti-periplakin, anti-desmoplakin, anti-BP180, and anti-BP230 Ig (76).

Recently, a novel lateral flow immunoassay (LFIA) was developed (82). It detects anti-Dsg 3 IgG in human sera. In contrast to other diagnostic procedures, the assay is simpler and faster. LFIA was validated on a collection of 200 sera and showed a sensitivity and specificity of 78.1 and 97.1%, respectively (82).

## Treatment

**Figure 5:**
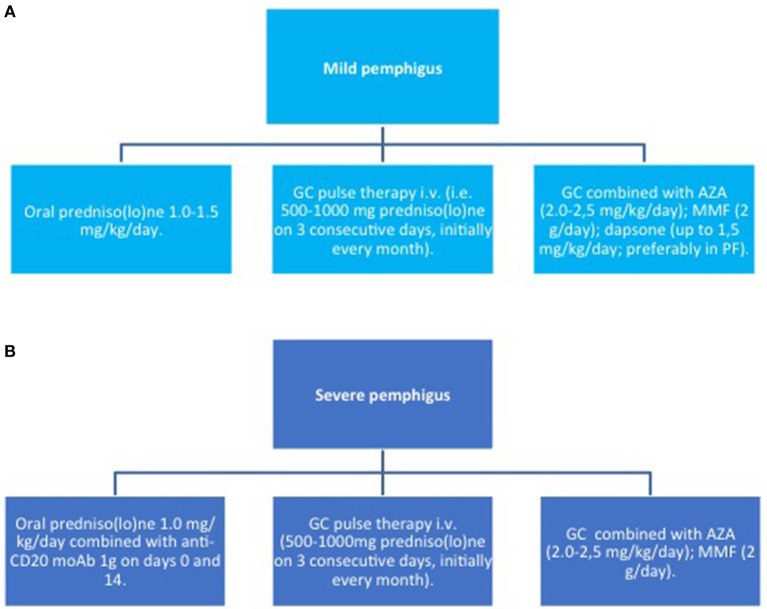
Induction therapy in pemphigus. **(A)** mild pemphigus; **(B)** severe pemphigus. AZA, azathioprine; GC, glucocorticoids; MMF, mycophenolate mofetil; moAb, monoclonal antibody.

**Figure 6:**
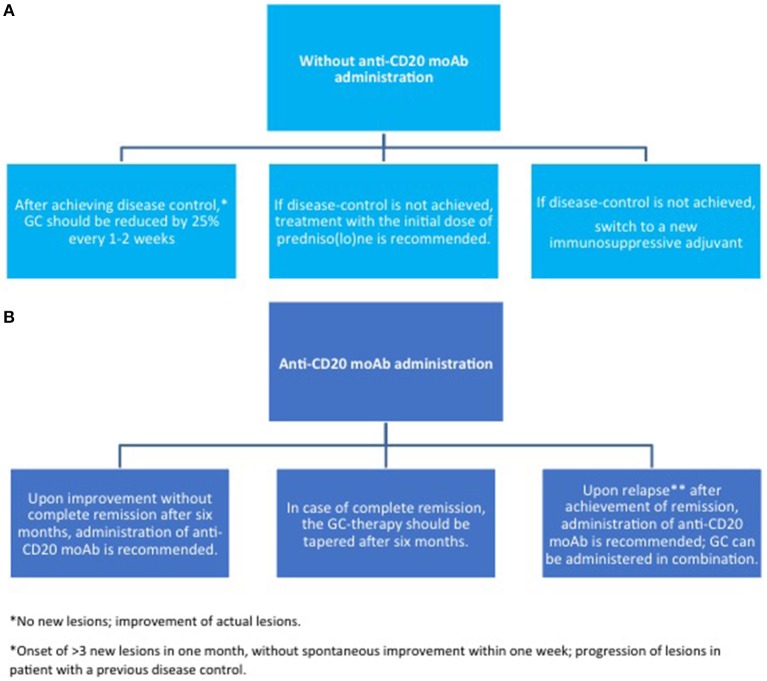
Maintenance therapy in pemphigus. **(A)** without anti-CD20 moAb; **(B)** with anti-CD20 moAb. GC, glucocorticoids; moAb, monoclonal antibody.

**Figure 7:**
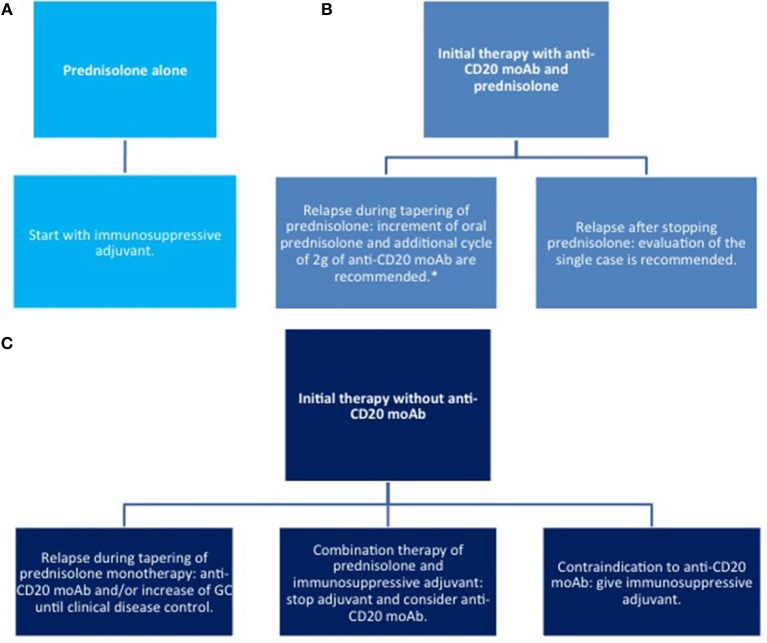
Therapy of relapse. **(A)** with corticosteroids only; **(B)** systemic corticosteroids combined with anti CD20 moAb; **(C)** systemic corticosteroids combined with other immunosuppressive agents. moAb, monoclonal antibody.

Since the advent of targeted therapies, the management of pemphigus has gradually changed. Until now, systemic corticosteroids (CS) and immunosuppressants have been the mainstay of pemphigus therapy. Among conventional adjuvant immunosuppressants, both EADV and BAD guidelines suggest azathioprine (AZA) and mycophenolate mofetil (MMF) as a first line steroid-sparing agent (83, 84). However, different variables, including patients' comorbidities, single institutional experience and costs have to be taken into account, and other drugs, such as methotrexate and cyclophosphamide, also demonstrate efficacy. Notably, these drugs have mainly a CS-sparing rather than a morbostatic effect (85–87). Accordingly, they do not lead to an improvement in achieving remission, but reduce the risk of relapse by 29% in comparison to CS alone (85). A recent prospective multicentre study by Joly et al. (88), now supports using RTX as a first line adjuvant therapy for pemphigus, showing superior efficacy compared to CS alone and reduced incidence of CS-related serious adverse events and overall mortality. The administration of intravenous immunoglobulin (IVIg) or immunoadsorption (IA) is a therapeutic option in patients with severe/refractory PV. Proposed algorithms for the induction and maintenance therapy as well as therapy of relapse are summarized in Figures 5–7.

### Corticosteroids and Immunosuppressants

#### Corticosteroids

In pemphigus, prednisolone is recommended as a first-line therapy in combination with an immunosuppressive agent, such as azathioprine (AZA) and mycophenolate mofetil (MMF), or RTX (83, 84). In addition, prednisolone alone at a dose of 1–1.5 mg/kg/day is still recommended as first line therapy in patients who are not eligible for treatment with RTX or other immunosuppressive adjuvants.

Higher CS doses (up to 1.5 mg/kg) may be administered, if disease control is not achieved within 3 weeks. As soon as disease control is reached, the doses should be tapered by a 25% reduction every other week. If lesions reappear, CS should be increased until two steps back at the previous dose to lead to disease control (84). However, the optimal dose has not been validated by randomized clinical trials RCT.

No significant differences regarding the duration of remissions and relapse rates have been reported in PV patients receiving low-dose oral prednisolone (45–60 mg/day) or high-dose oral prednisolone (120–180 mg/day) (89). In the maintenance period of PV, no advantages in terms of remission, death, relapse or withdrawal rates have been reported in patients on a pulsed CS therapy in comparison to conventional oral CS therapy. Indeed, the two RCTs about this topic reported opposite results (90, 91).

If the required CS dose is higher than 100 mg/day, a pulse therapy should be considered, in order to reduce the risk of adverse effects (92). Still, the advantage of combined oral CS pulses and immunosuppressive adjuvants is under debate (90, 93).

CS increase the expression of anti-inflammatory proteins and inhibit the production of pro-inflammatory proteins interacting with the cytoplasmic corticosteroid receptor (94). Indeed, the corticosteroid receptor reduces the expression of transcription factors as well as their co-activator molecules, such as NF-κB and activator protein 1 (94). In addition, CS induce the downregulation of IL-2, leading to a reduction in both B-cell clone expansion and autoantibodies synthesis (94). Furthermore, the reduction IL-2 expression inhibits the cell-mediated immunity and reduces T-cell proliferation (95). Therefore, CS lead to multiple signal transduction pathways producing anti-inflammatory, immunosuppressive, antiproliferative, and vasoconstrictive effects.

Several adverse effects have been described in patients undergoing long-time CS therapy, including increased overall susceptibility to infections and infestations, secondary adrenal insufficiency, osteoporosis, transient hyperglycaemia, hypertension, and posterior subcapsular cataract (96). In addition, cutaneous adverse effects have been described, including purpura, telangiectasias, atrophy, striae rubrae, acneiform or rosacea-like eruptions, infections, stern obesity and facial oedema.

#### Immunosuppressive Adjuvants

##### Azathioprine

AZA is a prodrug that converts to 6-mercaptopurine after oral administration. AZA down-regulates purine metabolism leading to a block of DNA, RNA and proteins synthesis. Furthermore, AZA inhibits mitosis and leads to immunosuppression in several ways (97). AZA reduces the number of monocytes and Langerhans cells, decreases γ-globulin production, and lower T-cell as well as suppressor B cell activity. Furthermore, it blocks T-helper-cell dependent responses of B cells (97, 98). 6-mercaptopurine can be inactivated to 6-methyl-mercaptopurine by thiopurine methyltransferase (TPMT) enzyme (96).

AZA is a safe CS-sparing agent, recommended as a first-line adjuvant immunosuppressant (83). The dosage of AZA is adapted to the TPMT activity and measurement of TPMT activity should be performed before AZA administration (99). Usually, 2.0 mg AZA kg/day are recommended by normal TPMT activity, while 1 mg AZA kg/day is recommended for patients with TPMT enzyme mutations (84). A dose of 50 mg AZA per day is recommended as initial therapy; the dose can be increased to the optimal dose based on TPMT activity (84). Adverse effects have been reported in 15–30% of patients. Severe adverse effects include myelosuppression and pancytopenia, and hepatotoxicity (99). However, myelosuppression may occur despite normal TPMT. Therefore, despite normal TPMT activity, a routine complete blood count including liver enzymes throughout the treatment period should be performed (99). In addition, long-term immunosuppression raises the risk of infections and cancer (100). Indeed, AZA shows a mutagenic potential that might provoke hematologic malignancies (100). Therefore, AZA is not recommended in pregnancy and breastfeeding (101). Other adverse effects include nausea, pancreatitis, diarrhea, aphthous stomatitis, maculopapular rashes, and anaphylaxis (99).

In a RCT involving 120 PV patients, a combined therapy of CS and adjuvant AZA (2.5 mg/kg daily) showed a higher CS-sparing effect than CS alone and a combination therapy with MMF (102). Furthermore, in a previous RCT study, adjuvant AZA (2.5 mg/kg daily) was compared to CS alone (103). During 1-year follow-up, a significant CS-sparing effect has been shown only in the last 3 months. Furthermore, disease activity was also significantly lower in the AZA group only in the last 3 months in comparison to the CS only. In addition, in a non-randomized study on PV patients, high-dose oral prednisone daily (1.5 mg/kg/day) vs. low-dose oral prednisone (40 mg every other day) plus AZA (100 mg/day) have been compared. It was shown a shorter main time to remission in high-dose oral prednisone monotherapy group, although the rate of adverse effects was higher (104). In summary, there is good evidence for a higher CS-sparing effect of AZA than CS monotherapy and MMF (83).

##### Mycophenolate mofetil

MMF is a prodrug that converts to mycophenolic acid (MPA) upon oral administration. MPA downregulates the immune system by selective impairment of inosine monophosphate dehydrogenase, leading to a blockade of the *de novo* pathway of purine synthesis in T and B cells, affecting both cellular and humoral immunity. Because lymphocytes are mainly dependent on the *de novo* pathway for purine biosynthesis, lymphocytes are the primary target of MPA. Because this target profile, MMF shows a safer profile in comparison to other less selective immunosuppressants, such as AZA (105).

As AZA, MMF is also recommended as a first-line adjuvant immunosuppressant (83, 84). The recommended dose of 2 g/day divided in two doses. In patients with a reduced renal function a reduced dosage should be administered (106). Initially, a dose of 500 mg MMF/day should be administered and an increase by 500 mg may be possible. A final dose of 2 g/day has been proposed in order to reach a better gastrointestinal tolerance (97).

Severe adverse effects have been rarely reported. Mild gastrointestinal symptoms, such as nausea, vomiting, and diarrhea are commonly seen. Rare are opportunistic infections, hematologic abnormalities, esophagitis, and gastritis (107). Studies on transplant recipients in therapy with MMF have demonstrated an increased risk of developing lymphomas and skin cancer (86). MMF is not recommended in pregnancy and breastfeeding, because of an increased risk of spontaneous abortion and congenital malformations (101).

Beissert et al. (108) compared CS plus MMF with CS plus placebo in PV patients. It has been highlighted that the MMF group showed a faster response to therapy, a longer disease-free interval, and a statistically significant CS-sparing. However, adjuvant MMF was not superior to CS in inducing disease control (108). Furthermore, no significant differences in cumulative CS dose, efficacy, or adverse effects were reported between AZA plus oral methylprednisolone and MMF plus oral methylprednisolone (109). In addition, Chams-Davatchi et al. (102) showed no significant difference in efficacy or safety between a combination therapy of prednisolone plus MMF (2 g/day) and prednisolone plus AZA. Regarding the CS-sparing effect, MMF was superior to prednisolone alone, but inferior to AZA, while compared to Cyclophosphamide (CYP), non-conclusive data were reported (102). The optimal MMF dose in PV has not yet been found. In a multicentric randomized controlled trial (RCT), no clear conclusions regarding the use of standard MMF dose (2 g daily) vs. a high one (3 g daily) were reported (108).

##### Cyclophosphamide

CYP is an alkylating prodrug with antineoplastic and immunosuppressive properties. CYP is converted in the liver into two active metabolites, phosphoramide mustard and aldophosphamide, which downregulate DNA replication and induce cell death. CYP shows also a blocking activity on proliferation, cytokine production, and lymphocyte-induced inflammation (85, 92).

The recommended oral dose is 2 mg/kg/day (97). Because of its rather unfavorable safety profile, CYP is not recommended as a first-line CS-sparing agent but rather as a rescue drug.

Several frequent adverse effects have been reported, including nausea, vomiting, diarrhea, hyperpigmentation of the skin/nails, and alopecia. Leukopenia, anemia, and thrombocytopenia may also occur. A severe complication is haemorrhagic cystitis, which may be prevented by adequate fluid intake and sodium 2-mercaptoethane sulfonate.

CYP shows a carcinogenic and teratogenic activity (86). As a result CYP administration is not allowed in pregnancy and breastfeeding (101). Moreover, temporary or permanent gonadal dysfunction has been described.

Three RCTs evaluated the CS-sparing effect of CYP. Chrysomallis et al. (110) compared oral CS monotherapy with a combined therapy of oral CYP and CS as well as a combined therapy of cyclosporine and CS. No difference in efficacy between these treatments were observed, but adverse events were higher in patients on combination treatment. An RCT comparing intravenous CYP pulse therapy (15 mg/kg monthly) plus CS vs. CS alone showed no conclusive difference in remission and relapse rates, cumulative steroid doses, and adverse events (111). Moreover, RCT regarding CYP pulse therapy (1 g monthly for 6 months, then 1 g every 2 months) plus CS in comparison to prednisolone alone and in combination with adjuvant AZA or MMF showed inconclusive results regarding efficacy and CS-sparing effect (102). Finally, oral methylprednisolone (2 mg/kg/day) combined with AZA (2–2.5 mg/kg/day) and a pulse CYP treatment protocol (500 mg intravenous CYP in combination with 100 mg intravenous dexamethasone for 3 consecutive days) were evaluated in a multicentric prospective RCT and did not show significant differences (112).

Different dexamethasone–CYP pulse therapy regimens were evaluated in two RCTs. In the first study, a combination of dexamethasone i.v. (100 mg on three consecutive days per months), CYP i.v. (500 mg once a month), and oral CYP (50 mg/day) was compared with CYP pulse therapy (15 mg/kg monthly) and prednisolone. In the first group a cutaneous, but not mucosal, response was faster achieved, while in the second group, the remission was seen earlier, but more severe CS-related adverse effects were reported (113). In the second study, patients under oral CYP alone (50 mg daily) and under a combination of dexamethasone i.v. (100 mg on three consecutive days monthly) with CYP i.v. (500 mg monthly) or oral CYP (50 mg daily on days between the pulses) were evaluated. No significant differences were reported regarding relapse rate, anti-Dsg1 and anti-Dsg3 autoantibodies titres, and the presence of tissue-binding autoantibodies by DIF (114).

##### Dapsone

Dapsone is used alone or in combination with topical clobetasol as first-line therapy in mild PF. Evaluation of serum glucose-6-phosphate dehydrogenase (G6PD) activity is mandatory before administration. The role of DA in the maintenance phase of PV has been evaluated only in one RCT, showing no statistical significance (115).

##### Methotrexate

Methotrexate (MTX) (10–20 mg/week) is considered a third-line CS-sparing drug in PV (83). In a retrospective single-center study, it has been reported that >80% of PV patients were able to reduce CS after 6 months on adjuvant MTX (15 mg/week) (116). Furthermore, Tran et al. (117) reported in a retrospective single-center study that 70% of PV patients stopped completely CS, mainly after 18 months. These findings support the concept that MTX has a CS-sparing effect in PV.

##### Cyclosporine

There are only limited data regarding the adjuvant use of cyclosporine in PV. Chrysomallis et al. (110) reported an inconclusive effect of adjuvant cyclosporine and a higher incidence of toxicities in combination treatment with prednisolone. Ioannides et al. (118) showed no advantage of this adjuvant drug over treatment with CS alone. Based on this data, CS is not recommended as adjuvant therapy in PV by the EADV or BAD guidelines (83, 84).

### Rituximab

#### Rituximab—Mode of Action

**Figure 8:**
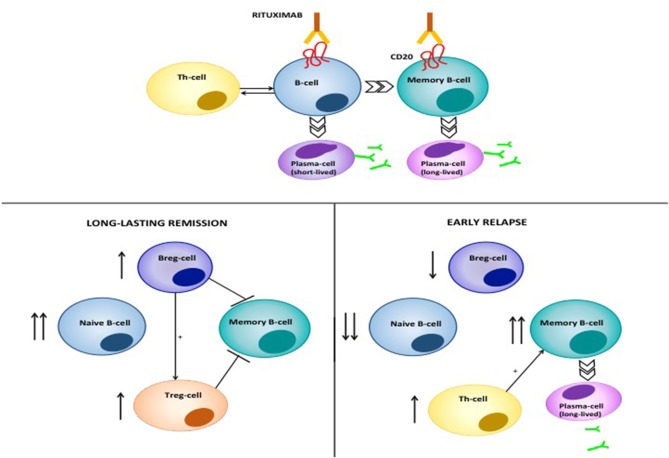
Mode of action of RTX in pemphigus. RTX induces depletion of B-cells and lymphoid resident memory B-cells by different mechanisms, including direct cell apoptosis, complement-dependent cytotoxicity and antibody-dependent cytotoxicity. The latter consists of the phagocytosis of opsonized B-cells by neutrophils, monocytes and macrophages, which express the Fcγ Receptor. Moreover, RTX significantly decreases T-cell function, by depleting antigen presenting B cells. Patients achieving durable responses have an increased naïve/memory B-cell ratio. Regulatory B-cells (B-regs) and regulatory T-cells (T-regs) are also increased, and are inhibitory on Dsg3-specific memory B-cells. On the contrary, patients with early relapses following B-cell repopulation have a decreased naïve/memory B-cell ratio. Reappearance of auto-reactive, Dsg3-specific T-cells contributes to activation of autoreactive B-cells and subsequent anti-Dsg IgG production.

The putative mode of action of RTX in pemphigus is shown in Figure 8. RTX is a chimeric type I monoclonal anti CD20 antibody, consisting of a human Fc portion and a murine variable region which serve as CD20 binding site (119). RTX was first licensed for use in B-cell malignancies (120), however, it is currently used in several autoimmune disorders, and has been recently licensed as first-line treatment in pemphigus (121–124). RTX target, CD20, is a transmembrane receptor, that is expressed across various developmental stages of the B-cell, from the pre-B cell to the mature; while, early precursor pro-B cells and antibody-producing plasma cells do not express it (125). Probably, it functions as a Ca^2+^ channel, that regulates intracellular Ca^2+^ influx through interaction between the intracytoplasmic domain and the activated B-cell receptor (119, 126). Noteworthy, CD20^−/−^ mice display normal B-cell development and function, and no enhanced susceptibility to infections (127). RTX binds near the large extracellular loop of CD20 (128). RTX binding to CD20 induces B-cell depletion by, at least, four different mechanisms: (i) direct induction of programmed cell death, which is dependent on activation of caspases and involves intracellular molecules, including Src kinases, p38 MAPK and NFkB (129–131); (ii) complement-dependent cytotoxicity, that happens when C1s binds to RTX opsonized cells and triggers complement activation and formation of the membrane attack complex (MAC), which eventually induces cell lysis (132); (iii) antibody-dependent cytotoxicity, which consists of activation of NK cells through binding the human Fc portion of RTX to the FcRIII receptor: this activates NK cells to release cytotoxic mediators, including perforins and granzyme B, which induces caspases-dependent cell death in the target lymphocyte (133); (iv) antibody-dependent phagocytosis, in which neutrophils, monocytes and macrophages bind RTX opsonized B-cells through the Fcγ Receptor (132). Recently, a new mechanism, referred to as trogocytosis, or shaving, has been characterized. In trogocytosis, macrophages remove RTX-CD20 complexes by transferring plasma membrane; this triggers cell death through a yet-to-be identified mechanism (134).

RTX is a highly effective therapy in pemphigus (1). By depleting B-cells, RTX leads to marked decrease of circulating anti-Dsg autoantibodies, and, since the pathogenic role of such autoantibodies, significant amelioration of the lesions (135). Multiple lines of evidence, however, suggest that RTX exerts a deep modulation of both humoral and acquired immune function in pemphigus, explaining the fact that, in many cases, disease amelioration lasts longer than B-cell re-appearance in the peripheral blood of the patients (136). First, it should be noted that RTX, in parallel with a drastic decrease of pathogenic autoantibodies, induces drop of total serum IgM, but does not modify total serum IgG, thus suggesting that long-lived plasmablasts accounting for antibody production against microbes are not affected (137). Previously, we showed that in PV patients, RTX induced elevation of B-cell activating factor (BAFF) in parallel with decreasing pathogenic antibody levels and increased IgG titer against Varicella Zoster virus and Epstein Barr virus: thus elevation of BAFF may at least partly exerts a stimulatory role on long-lived plasma blasts (124). Likewise, one study recently showed that while autoimmune blistering disease patients receiving RTX showed reduced circulating memory B-cells against the influenza virus compared to healthy people, they showed comparable recall response to vaccination, suggesting the existence of a memory B-cell compartment, probably resident in lymphoid tissues, which is not depleted by RTX (138). Interestingly, when B-cells re-appear following RTX treatment, there is a substantial increase in the proportion of naive and transitional B-cells and an increased naive/memory B-cell ratio (137). Patients in complete clinical response also display increased number of IL-10-producing regulatory B-cells and absence of Dsg3-IgG^+^ B-cells (136). Altogether, these findings suggest that RTX induces a complete reset of the B-cell repertoire in pemphigus, favoring early appearance of immature B-cells and anti-inflammatory regulatory B (Breg) cells, and delayed reappearance of Dsg-specific memory B-cells, which eventually account for disease relapse (136, 139).

Our group demonstrated that, besides pleiotropic effects on B-cells, RTX inhibits auto-reactive Th1 and Th2 cells, by interfering with the T-B cell cross-talk, in which Dsg-specific B-cells probably serve as antigen presenting cells. Decreasing Th1 and Th2 functions occurred early following RTX and lasted around 6 and 12 months, respectively. Conversely, we did not observe inhibition of Dsg-specific regulatory T (Treg)-cells, which account for maintaining peripheral tolerance against Dsg antigens. Finally, we demonstrated that T-cells specific for the tetanus toxoid as well as the total count of CD3^+^CD4^+^ T cells were not decreased by RTX (140). Similar results were found in a subsequent study by Leshem et al. (141), confirming that RTX impairs autoreactive, rather than global T-cell function in pemphigus.

RTX side effects mostly include infections and infusion-related adverse events. In fact, while single RTX infusions may not impair significantly memory responses against previously encountered pathogens (138), patients mounts a defective immune reactions against newly encountered pathogens and serious and life-threatening infections, including sepsis, following RTX treatment have been variously reported (120, 142–147). Opportunistic infections can also occur, including cytomegalovirus and *Pneumocytic jiroveci* infections (148–153). In this regard, it is not known yet whether pemphigus patients receiving RTX may benefit from prophylaxis against *Pneumocystis jiroveci* infection (154). A theoretical risk of reactivation of hepatitis B and C viruses as well as tuberculosis should be also taken into account (138, 155–157). Infusion-related adverse events mostly occur during the time of infusion and include type I hypersensitivity reaction and anaphylaxis, and cytokine release syndrome (158–162), although the latter has never been reported in pemphigus patients (142, 163). Delayed reactions include serum sickness, vasculitis and Steven Johnson syndrome (164–167). Interestingly, there have been some cases of paradoxical pemphigus flares following RTX treatment. In one case, paradoxical pemphigus flare was accompanied by increased serum concentrations of anti-Dsg3 IgG autoantibodies (168). In any case, disease flare predicted treatment failure. Suggested underlying mechanisms include increased cytokine release from apoptotic B-cells, immediate depletion of regulatory B-cells or a transient lymphocyte activation following RTX-CD20 interaction (141, 163, 168–171).

There is much controversy about the optimal RTX dose in pemphigus. Two main protocols are used: the rheumatoid arthritis protocol, which consists of two 1,000 mg infusions 2 weeks apart, and the lymphoma protocol, which consists of four 500 mg infusions once a week (135). There are not yet randomized trials assessing which protocol is better in terms of efficacy and safety. On the other hand, high dose regimens should be preferred instead of low-dose regimens, due to longer disease response (163). Nowadays, one study recently reported on a pemphigus patient achieving successful disease remission with an ultra-low dose of RTX (200 mg in a single infusion), with persistent B-cell depletion after 6-month follow-up (172, 173). This highlights the need of further investigating individual factors that may influence RTX efficacy in an effort to personalize treatment schedules and optimize the safety.

#### Rituximab in Pemphigus Vulgaris and Pemphigus Foliaceus

RTX was initially shown to be effective for pemphigus patients resistant to standard immunosuppressive therapies. In one study in 2006, Ahmed et al. (174) reported complete clinical remission, allowing successful tapering of immunosuppressive therapies, in 9 out of 11 patients with refractory pemphigus following a protocol combining IVIg and 10 RTX infusions over a 6 months period (Table 1). After 10-year follow-up most patients were shown to maintain clinical remission (175). In 2007, in a larger clinical series, Joly et al. (176) reported durable clinical response with significant corticosteroid sparing effects in 86% of 21 patients with refractory pemphigus following a single cycle of RTX. Similar results were reported in a series of 42 patients by Cianchini et al. (177), they also observed that a recall infusion of 500 mg was effective for patients who relapsed following initial disease control. One study including 136 patients with refractory pemphigus from four different European countries reported a 95% overall response rate, with two third of patients achieving complete remission (178). Likewise, review articles and meta-analysis estimated complete clinical remission occurring in 76–90% of patients within a median time of ~6 months (146, 163). Mean remission duration ranged from 15 to 17 months, although only a small percentage of patients maintained remission off therapy (146, 163). In two studies, RTX use early in the disease course resulted in significantly higher and longer clinical response (136, 179). The same finding emerged also in a study by Amber and Hertl, reviewing clinical outcomes of 155 pemphigus patients treated with a single cycle of RTX (179, 180). These preliminary observations have lead different research groups to investigate the potential benefit of RTX applied as a first line therapy in pemphigus. Indeed, first-line therapy with RTX, combined either with high potency topical corticosteroids or IVIg, was shown to be effective in pemphigus patients with contraindication to systemic steroids (181, 182). One retrospective study found significantly higher rate of complete remission off immunosuppressive therapy in patients who were administered RTX as a first-line steroid sparing agent compared to patients who received RTX after failing other immunosuppressants (183). In 2017 a large prospective randomized trial, comparing RTX combined with a short course of prednisone vs. prednisone alone in patients with newly diagnosed pemphigus, demonstrated significantly higher complete remission rate off therapy in patients receiving RTX, resulting in a dramatic decrease of the cumulative steroid dose and significantly fewer adverse events. Furthermore, re-treatment with a single RTX dose of 500 mg after 12 and 18 months was highly effective and well-tolerated in achieving long-term clinical remissions (88). Interestingly, also patients with PF, in whom the rate of remission with RTX was estimated around 50% in the refractory setting, were shown to respond well to RTX when applied as a first line therapy (88, 184–186). In summary, while RTX was initially recommended as a third line therapy in patients without adequate disease control with standard immunosuppressants, several studies have definitively demonstrated that patients may benefit from early RTX treatment, in terms of both clinical efficacy and safety, leading current guidelines to recommend it as the gold standard for new onset pemphigus (187, 188).

#### Rituximab in Paraneoplastic Pemphigus

PNP usually occurs secondary to B-cell neoplasms, hence RTX appears to be a reasonable treatment, targeting both autoreactive and malignant B-cells (189–191). Several cases in the literature have shown remarkable responses in B-cell malignancies-associated PNP using RTX either alone or in combination with immunosuppressants or chemotherapy (192–195). However, the overall efficacy of RTX in PNP is much less consistent than PV and PF (196); mucosal lesions were shown to be particularly resistant to RTX treatment (197). Moreover, some authors pointed out that RTX treatment of the underlying malignancy may paradoxically trigger PNP (198).

PNP combines clinical and histologic features of PV and lichenoid/interface dermatitis, reflecting a mixed B- and T-cell response against epidermal autoantigens. Indeed, while declining B-cells autoreactivity, RTX may be ineffective against clinical manifestations secondary to auto-reactive T-cells activation (199). Indeed, RTX use to treat the underlying malignancy may lead to overlook the diagnosis of PNP in patients presenting with lichenoid dermatitis or toxic epidermal necrolysis-like lesions without detectable circulating autoantibodies and negative DIF. This was recently hypothesized in an interesting study by Kwatra et al. where the authors observed a significant reduction in the cases of B-cell lymphoma-associated PNP from 2011 to 2017 compared to the period from 2003 to 2010. All the patients diagnosed with PNP during or after 2011 had already received RTX; whereas, most of the cases before 2011 did not (199).

### Is There Evidence for a Maintenance Therapy With Rituximab in Pemphigus?

Relapse following RTX occurs in about 40–80% of the patients (163), within a mean time ranging from 6 to 24 months (153, 200). Additional cycles of RTX were shown to be effective in relapsed patients, suggesting that patients may benefit from maintenance with RTX (88, 176, 177). However, the exact timing of RTX re-treatment to prevent relapse is uncertain (187). Re-treatment at 6 months has been adopted empirically, but it is not currently validated in the setting of randomized trials (201). Noteworthy, there is a subset of patients who achieves long-lasting remission even with a single cycle of RTX (136, 176, 202). In these patients, additional prophylactic cycles of RTX not only result in unneeded costs, but also substantially increase the risk of adverse events. Developing biomarkers that could identify patients at higher risk of relapse following RTX is therefore an urgent need.

Relapse following RTX can be attributed to persistence of autoreactive B-cells, because of incomplete B-cell depletion, or re-appearance of Dsg-specific B-cell clones during B-cell repopulation. Thus, monitoring the B-cell repertoire appears to be a suitable tool to predict the risk of relapse following RTX. In one study, shorter time to relapse was found in patients receiving adjuvant immunosuppressants during and following RTX treatment, suggesting a possible effect of prolonged immunosuppression on immunosurveillance, favoring an early re-appearance of autoreactive B-cells (203). In 2008, a consensus of German experts recommended checking the number of CD19^+^ B-cells in the blood at baseline and after RTX treatment (204). In one retrospective study by Albers et al. (200) including 62 pemphigus patients treated with a total of 99 RTX cycles, the number of CD19^+^ B-cells were shown to be a useful predictor of relapse. A time to B-cell repopulation lower than 12 months also correlated with the risk of relapse. However, relapse has reportedly occurred even before B-cell re-population (205). In these cases, it is conceivable that lymphoid tissues served as a reservoir for autoreactive B-cells and protected them from RTX.

Longitudinal analysis of the naïve/memory B-cells ratio and the number of B-regs may also provide useful information (206). Interestingly, Albers et al. (200) found an inverted correlation between CD4^+^ T-cell counts following RTX and the risk of relapse. Autoreactive T-cells are essential in pemphigus to orchestrate the B-cell responses and autoantibody production against Dsgs. RTX also is effective in decreasing peripheral T-cell response against Dsgs (140). Thus, high CD4^+^ T-cells count would be expected to predict relapse. However, Albers et al. (200) speculated that the protective role of CD4^+^ T-cells could be attributed to Treg cells, which prevented further expansion of autoreactive B-cell clones. Levels of circulating anti-Dsg autoantibodies were also shown to be involved in the relapse of patients with pemphigus (207). In Albers et al. (200), levels of anti-Dsg3 IgG were predictive of relapse in patients with mucocutaneous and mucosal disease, whereas anti-Dsg1 IgG were predictive for the subset of mucosal PV patients with cutaneous involvement. However, in other studies, elevation of anti-Dsg3 IgG was also noted in patients maintaining a clinical remission, suggesting that in some cases anti-Dsg3 IgG may target non-pathogenic epitopes of the Dsg3 ectodomain (208). Mouquet et al. (137) found that elevation of Dsg1 autoantibodies was associated with early relapse following RTX.

In a prospective study, a high baseline index of anti-Dsg1 IgG was found in early relapsing patients compared to late relapsing patients following RTX. Baseline anti-Dsg1 IgG, but not Dsg3-IgG, indeed showed a significant positive correlation with a risk of relapse within 12 months after RTX treatment, thus suggesting that patients with high anti-Dsg1 IgG before treatment deserve a close monitoring during the 12 months following treatment, or at least may benefit from a prophylactic RTX dose during the first 12 months. Also in this study, later B-cell repopulation was found in patients experiencing a late relapse compared to patients experiencing an early relapse (209). By contrast, in a retrospective study including 40 pemphigus patients treated with RTX, mucosal involvement was found to be associated with a poor clinical outcome and relapse (210).

### Intravenous Immunoglobulin

IVIg consist of human plasma-derived IgG, sugars, salts and solvents. IVIg derived from large plasma pools. Albeit not immunosuppressive, they exerts various anti-inflammatory effects, including Fc receptor blockade, stimulation of antibodies production against different subclasses of T lymphocytes, inhibition of different T-cell functions, complement hindrance via inactivating C3 precursors, dendritic cell downregulation, B-lymphocyte apoptosis, inhibition of phagocytosis, and increment of response to steroids (211). However, the main mode of action is an increased catabolism of immunoglobulins via binding to the neonatal Fc receptor (FcRn) (211).

A dose of 2 g per kg body weight per treatment cycle is recommended. High-dose IVIg was shown to independently increase disease control in pemphigus (85). IVIg is mostly used as an adjuvant therapy to CS and immunosuppressive drugs in recalcitrant PV. Indeed, Amagai et al. (212) reported that high dose IVIg (0.4 g/kg per day) over 5 days resulted in significantly reduced disease activity and autoantibody titres in 51 patients with CS-resistant PV. Furthermore, Svecova et al. (213) reported a significant improvement in Pemphigus Disease Area Index (PDAI) and a reduction of 90% of the CS dose in a cohort of 10 CS-resistant PV. Combination of IVIg and RTX also demonstrated efficacy (Table 2).

**Table 2:** Combination therapy of rituximab with intravenous immunoglobulins (IVIg) or immunoadsorption (IA).

**Study**	**Study design**	**Synopsis**	**Adverse events**
**RTX PLUS IVIg**			
Ahmed et al. (174)	Prospective, including 11 refractory PV patients, treated with 2 cycles of RTX 375 mg/Kg/m^2^ once a week for 3 weeks followed by a cycle of IVIg 2 g/Kg in the fourth week. Maintenance with RTX and IVIg infusions once a month for 4 months	9 out of 11 patients achieved a complete remission in parallel with a rapid decreasing of the serum concentrations of anti-Dsg autoantibodies, which allowed successful discontinuation of steroids and adjuvant immunosuppressants. Clinical responses lasted 22–37 months (follow-up after discontinuation of RTX: 15–37 months). In 2 patients experiencing a relapse, retreatment with RTX was effective	No relevant side effects reported, including infections or infusion-related reactions
Ahmed et al. (175)	10-year follow-up study of Ahmed et al. (174)	All the 10 patients previously treated with RTX and IVIg retained clinical remission after 10 years from the last RTX infusion	Not observed
Feldman et al. (205)	Retrospective, including 19 patients with refractory pemphigus. RTX was given at week 1, 2, 3, 4, 5, 6, 7, 8, 12, 16, 20, 24. IVIg were given at week 0, 4, 8, 12, 16, 20, 24	11 patients achieved long-term remission, allowing discontinuation of corticosteroids and other immunosuppressants. 8 suffered at total of 15 relapses. Re-treatment with RTX and IVIg was effective in achieving a long-term remission. Relapse was associated with incomplete B-cell depletion, B-cell repopulation and raise of serum anti-Dsg autoantibodies	Not reported
**RTX PLUS IA**			
Behzad et al. (208)	Retrospective, including 10 patients with refractory PV. IA was administered at 4-week intervals, followed by RTX according to either the lymphoma protocol or the RA protocol	8 out of 10 patients obtained a complete remission on therapy at 6 months following the first IA treatment. In 6 of them complete remission on therapy persisted at 12 months. Treatment with IA and RTX leads to a successful tapering of oral prednisone	No serious adverse events reported
Kasperkiewicz et al. (214)	Clinical series including 23 consecutive patients. IA was given on 3 consecutive days and repeated at initially 3 and then 4 weeks until lesions clearance of 90%. RTX 1,000 mg was infused at weeks 1 and 3. Patients also received intravenous dexamethasone pulses and oral azathioprine or mycophenolic acid	19 patients achieved long-term complete remission. Over a period of a mean of 29-month follow-up, 6 patients suffered a relapse	A *Staphylococcus aureus sepsis* associated with an infected central intravenous line and an episode of extensive herpes simplex infection
Kolesnik et al. (215)	Retrospective, including 4 patients with PV and 2 with PF. The treatment protocol included a combination of protein A IA and RTX (375 mg/m^2^ once a week the day after each IA session) (Magdeburg treatment protocol). Patients with sub-epidermal blistering dermatoses were also included	Complete or partial remission was observed in 88 and 12% of patients, respectively, within an average follow-up of 22 months. Relapse occurred in one patient with PF. Treatment was associated with a substantial decrease of serum autoantibody concentrations	Erysipelas at the lower leg of one patient due to trauma
**RTX PLUS IVIg PLUS IA**			
Shimanovich et al. (216)	Clinical series, including 5 patients with PV and 2 with PF. Treatment included a combination of protein A IA and RTX (375 mg/m^2^ once a week per 4 consecutive weeks). All patients received adjuvant immunosuppressive therapies. IVIg were given in non-responder patients	Long-term remission was achieved by 3 patients. Partial remission was induced in 1. Three refractory patients achieved long-term disease control following IVIg therapy	*Staphylococcus aureus* bacteremia, deep venous thrombosis and *P. carinii* pneumonia

*RTX, rituximab; IA, immunoadsorption; IVIG, intravenous immunoglobulins; PV, pemphigus vulgaris; PF, pemphigus foliaceus; RA, rheumatoid arthritis*.

Adverse effects have been reported in <5% of patients and occur more often in patients who are IVIg-naive or at risk of bacterial infections (217, 218). Immediate adverse effects (occurring within the first hour of infusion) include headache, nausea, fever, tachycardia, malaise, arthralgia, and dyspnoea. Delayed reactions include headache, acute renal failure, thromboembolic events, and pseudohyponatremia (218). Myocardial infarction, thrombosis, pulmonary embolus and Stevens–Johnson syndrome, have been also described. Thrombosis can be provoked by hypercoagulability due to increased blood viscosity, augmented fibrinogen production, and raised platelet activity (219). Therefore, high-risk patients, such as elderly or people affected by hypertension or coronary heart disease, should be screened appropriately and prophylactic anticoagulation need to be considered. In a series of 54 patients on IVIg treatment, an incidence of aseptic meningitis of 11% has been reported. Risk factors were a previous history of migraine and high-dose IVIg regimen (220). Finally transient acute kidney injury has been also described (221).

### Immunoadsorption

IA consists of the passive removal of IgG from the patient's systemic circulation. In IA, the blood is passed through adsorber columns, in which molecules with high affinity for IgG, i.g. protein A (Immunosorba®) or the synthetic peptide PGAM146 (Globaffin®), function as a ligand (135, 222). Basic principles of IA are, indeed, similar to plasmapheresis, but, compared to the latter, IA does not remove plasma proteins, such as albumin and clotting factors. The use of plasmapheresis in pemphigus has been largely abandoned due to significant incidence of serious adverse events, such as sepsis (223). The fact that IA does not require replacement of fresh frozen plasma and albumins allows processing higher plasma volume per treatment session, resulting in a lower, albeit not abolished, risk of adverse events. Nevertheless, infections are still the most frequently encountered complication and can occur either secondary to the IA procedure, i.g. cathether-associated infections, or secondary to decreased serum concentrations of protective antibodies (224–226).

IA is an ideal treatment for pemphigus patients with severe and extensive disease at baseline. Combining IA with immunosuppressive therapies provides faster clinical responses compared to the immunosuppressive therapy alone, since IA allows immediate removal of pathogenic antibodies, whose serum concentration reflects both disease activity and severity. Once circulating antibodies are removed, a positive gradient between the skin and blood leads skin-bound autoantibodies to move into the systemic circulation. To avoid a rebound increase of the autoantibody titer, IA is therefore performed on 3 or 4 consecutive days, and then repeated on a monthly base based on the disease response, autoantibody serum concentrations and treatment tolerability (227).

Current guidelines indicate IA as a reliable first-line treatment in pemphigus patients, in whom lesions cover (1) > 30% of the body surface or (2) > 25% of oral or genital mucous membranes or involve (3) the conjunctiva or (4) the esophagus; it can be also recommended in refractory patients with more than 3 months of active disease despite at least two immunosuppressive therapies (84, 187, 188).

Possibly, IA may also exert immunomodulating properties, which account for its synergistic effect in combination with RTX (Table 2). Accordingly, in a study by Amber and Hertl, IA was the only adjuvant treatment resulting in a lower risk of relapse following RTX (180). Indeed two studies, including one by our group, demonstrated rapid and durable clinical response combining RTX and IA with or without oral immunosuppressants (208, 214). Moreover, a yet unpublished German multicentric prospective randomized trial comparing IA plus the best medical therapy vs. the best medical therapy alone found that the adjunct of IA resulted in faster withdrawing prednisone and reduced cumulative steroid dose to achieve pemphigus remission (DRKS 00000566).

Langenhan et al. (228) developed specific adsorbers using Dsg1 and Dsg3 ectodomains as a ligand which were shown to efficiently remove pathogenic autoantibodies by 25 and 21%, respectively, without significant variation of anti-EBNA 1 IgG. The same group demonstrated that Dsg3/Dsg1 specific IA eliminated the capacity of PV sera to induce Dsg3 internalization *in vitro* and blistering in neonatal mice (229). Hopefully, future clinical application of these absorbers would lead to increase the safety of IA, reducing the incidence of infections secondary to hypogammaglobulinemia.

Double filtration plasmapheresis (DFPP) is a relatively new procedure that, similar to IA, removes selectively immunoglobulins, while minimizing the loss of albumin. In small case series and one retrospective study DFPP also demonstrated efficacy in drug resistant pemphigus (230–232).

## Emerging Therapies

### B-Cell Therapies Other Than Rituximab

**Figure 9:**
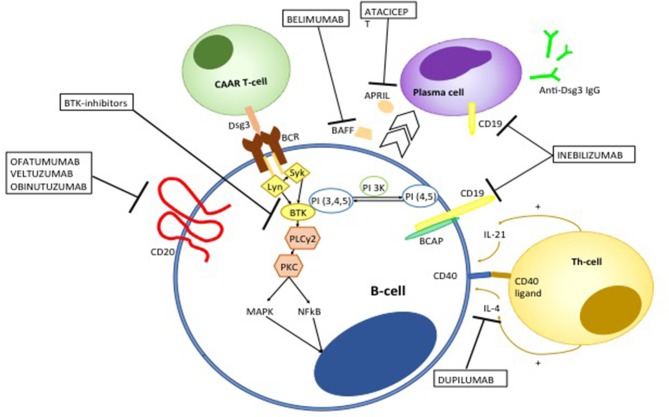
Emerging therapies targeting auto-reactive B and T-cells in pemphigus. Ofatumumab, veltuzumab, and obinutuzumab are fully human or humanized monoclonal antibodies targeting CD20. Ofatumumab and veltuzumab are class I anti-CD20 monoclonal antibody, with a higher capacity of binding CD20 and inducing complement-dependent cytotoxicity compared to RTX. Obinutuzumab is a class II anti-CD20 monoclonal antibody, that has an increased affinity to the FcγIII receptor, resulting in a more potent antibody-dependent cytotoxicity. Bruton kinase (BTK) inhibitors interfere with B cell activation. BCR signaling induces migration of BTK from the cytosol to the cell membrane, though the interaction with phosphatidylinositol 3,4,5-triphosfate generated by phosphoinositide 3-kinase (PI3K). BTK is activated by Lyn and Syk and then actives downstream molecules including phospholipase C gamma 2 (PLCγ2) and Protein kinase C. The latter in turns activate different pro-inflammatory pathways including mitogen associated protein kinases (MAPK) and Nuclear Factor k B (NFkB). Chimeric autoantibody receptor (CAAR)-T-cells are engineered T-lymphocytes which express Dsg3 ectodomain, which allows recognition and subsequent killing of B-cells targeting Dsg3. Belimumab and atacicept target B-cell derived B-cell activating factor (BAFF) and a proliferation-inducing ligand (APRIL), respectively, which promote differentiation toward autoantibody-producing plasma cells. Inebilizumab is a monoclonal antibody targeting CD19 which is not only expressed on B cells but also plasma cells. Dupilumamb is a monoclonal antibody targeting interleukin (IL-4), which is one the main cytokine produced by T helper 2 cells and T follicular helper cell which induces autoantibody production by autoreactive B-cells.

Although RTX has dramatically improved the overall prognosis of pemphigus, treatment failure or early relapse may be observed. RTX is a chimeric monoclonal antibody, whose murine component is thought to be responsible for the observed allergic reactions during the infusion. However, it also accounts for the appearance of human anti-chimeric antibodies (HACAT), which may potentially limit the efficacy of the drug (233). Over the recent years, different monoclonal antibodies targeting CD20 have been developed. Second generation anti-CD20 mAb differ from RTX in that they are humanized or fully human, taking advantage of being less immunogenic (234) (Figure 9). Amongst second-generation anti-CD20 monoclonal antibodies, ofatumumab has been the first to be approved. It is a type I anti-CD20 monoclonal antibodies that targets the extracellular portion of CD20 close to the B-cell membrane, resulting in a more potent complement-dependent cytotoxicity compared to RTX (235). Ofatumumab demonstrated efficacy in a patient with pemphigus, in whom RTX loses efficacy presumably because of the appearance of HACAT (236). Unfortunately, a randomized controlled trial of ofatumumab in pemphigus has been prematurely terminated due to financial restrictions (186). Similarly, Ellebrecht et al. (237) described successful treatment with subcutaneous veltuzmab in a pemphigus patient who only achieved a partial remission with RTX. Veltuzumab is a type I humanized anti CD20 monoclonal antibody that has similar complementary-determining regions of RTX, but a 2.7-fold greater binding avidity and effect on complement-dependent cytotoxicity than RTX. It can be also administered subcutaneously, resulting in lower side effects than intravenous RTX (234).

Obinutuzumab is a third generation glycoenginereed type II humanized anti-CD20 monoclonal antibody. Differently from type I monoclonal antibodies, obinutuzumab shows enhanced capacity to induce direct apoptosis (via a caspase-independent factor) and antibody-dependent cytotoxicity, whereas it does not induce complement-dependent cytotoxicity (238–240). Obinutuzumab has shown to induce superior B-cell depletion compared to RTX in blood samples from patients with rheumatoid arthritis and systemic lupus erythematosus. More intriguingly, it has been shown to induce significant cytotoxicicty also in naive and class-switched memory B-cells, a high number of which may be implicated in early relapse following RTX treatment in pemphigus (241). Other therapies of interest include belimumab and atacicept, a monoclonal human IgG1 antibody and a fully human recombinant fusion protein, which respectively, target BAFF and a proliferating-induced ligand (APRIL), which are involved in B-cell differentiation in antibody producing plasma cells (242, 243). It is worth mentioning that a monoclonal anti-BAFF-receptor antibody (VAY736) is being investigated in a randomized, partial-blind, placebo- controlled multicentre trial (NCT01930175) (125).

Bruton kinase (BTK) inhibitors are also a promising B-cell targeting therapy in pemphigus. BTK is a member of the Tec family of kinases, which is mainly expressed on B-cells, excluding antibody-producing plasma cells. Activation of BTK following antigen-recognition by the BCR activates different downstream molecules including p38MAPK, MEK/ERK, and NFkB, whose related signals are key regulator of B-cells survival, proliferation, maturation and antigen-presentation (244, 245). BTK inhibitors such as ibrutinib have shown impressive clinical responses in patients with B-cell malignancies, but also hold promise for the treatment of autoimmune disorders. In particular, over-activation of BTK have been shown to drive autoimmunity by enhancing autoantibody production and class switching, promoting B-T cell cross talk and peripheral B-cell loss of tolerance (244). Furthermore, enhanced expression of BTK in B-cells induces differentiation of T follicular helper cells, which have been shown to be involved in the pathogenesis of pemphigus (67, 246). Interestingly, ibrutinib has been successfully used in two cases of PNP associated with B-cell malignancies (247, 248). The efficacy of PRN1008, an oral inhibitor of BTK is currently being evaluated in a clinical trial (NCT02704429) (125, 249).

Monoclonal antibodies targeting CD19^+^ (a B-cell surface molecule which is also expressed on antibody producing plasma cells) such as inebilizumab, would be an effective strategy in pemphigus, since persistence of long-lived plasmablasts producing anti-Dsg IgG autoantibodies is presumably amongst the mechanisms of disease resistance to RTX treatment (125). Figure 9 summarizes how emerging anti-B-cell therapies works in pemphigus.

### Chimeric Autoantibody Receptor (CAAR)-T Cell: A Most Promising Treatment Approach in Pemphigus

Chimeric antigen receptor (CAR)-T-cell therapy has shown remarkable efficacy in otherwise untreatable hematologic malignancies (250). Currently, two CD19-directed CAR-T-cell therapy, tisagenlecleucel and axicabtagene ciloleucel have been approved for treatment of heavily refractory/relapsed acute lymphoblastic leukemia and B-cell aggressive lymphomas (251–254). The noteworthy antitumor activity of CAR-T cells in hematologic malignancies has recently led to investigate potential clinical application of such a therapy in solid tumors (255–257); an intriguing scenario has been also opened with regard to autoimmune diseases (258). CAR-T cell therapy represents an example of adoptive cell transfer therapy: patient's derived T-cells are modified *ex-vivo* to express a CAR, which allows selective recognition of the antigen of interest and consequent killing, via an MHC-unrestricted manner, of the antigen-bearing cells (259–261).

CARs are fusion proteins whose structure comprises three domains: (i) the extracellular domain, which consists of a single chain variable fragment and serves as antigen recognition domain; (ii) the transmembrane domain; and (iii) the intracellular domain, which consists of the zeta (ζ) chain of the CD3, a component of the endogenous T-cell receptor (262). In second and third-generation CARs, the intracellular domain is linked to co-stimulatory molecules, specifically 4-IBB and CD28, which promote survival and proliferation of CAR-T cells following antigen recognition, resulting in higher clinical efficacy (263). Production of CAR-T-cells requires different steps. Briefly, the CAR protein is cloned into lentiviral or retroviral plasmids. Viral vectors are then transfected to packaging cell lines, such as HEK293 cells, in order to obtain large amounts of the CAR-bearing plasmids. Patient's T-cells, which are obtained by leukapheresis, are incubated with the viral vector, which enters into the cells and introduces the CAR encoding-RNA. The latter is reverse-transcribed in DNA and stably integrates into the T-cell genome. The CAR protein can be then transcribed and translated and eventually expressed on the T-cell surface. CAR T-cells are finally expanded, concentrated and cryopreserved to be then re-infused into the patient (263, 264).

Recently, Ellebrecht et al. (265) created a “chimeric autoantibody receptor” (CAAR), whose extracellular domain consisted of Dsg3 fragments. T-cells engineered to express Dsg3 CAAR were shown to selectively target human anti-Dsg3 B-cells *in vitro*. Anti-Dsg3 antibodies derived from patients' sera did not abolish CAAR-T cells activity. In a PV murine model, CAAR-T cells reduced pathogenic IgG antibodies and ameliorated disease severity. Interestingly CAAR-T-cells were shown to target B-cells bearing antibodies against different Dsg3 epitopes, providing support for their efficacy in a disease typically characterized by oligo-clonality (265, 266). Apart from the possibility that Dsg3-CAAR T-cells could potentially target keratinocytes expressing desmocollins and desmogleins, which physiologically binds to Dsg3, the authors did not observe any significant toxicity (the so-called off-target toxicity) against keratinocytes. Finally, the authors reported similar activity against anti-Dsg3 B-cells between CAAR-T-cells and CD19^+^ CAR T-cells, hence suggesting that selective target of self-reactive B-lymphocytes does not result in reduced treatment efficacy (265).

A major advantage of CAAR-T cell therapy in PV involves the possibility to target memory B cells, which still accounts for the observed relapse following anti CD20 therapies (136, 267). Moreover, since part of CAAR-T-cells differentiates toward memory cells (267), this may prevent future formation and expansion of Dsg 3-reactive B-cells, conferring such a kind of “immunity against autoimmunity.” A second advantage of CAAR-T-cell therapy is reduced immunosuppression, since normal B-cells are not expected to be killed (265).

Since auto-reactive B-cells in pemphigus account for only a minor subset of total B-cells, it is unlikely that CAAR-T-cell therapy may lead to serious adverse events as has been observed in the onco-hematologic setting (265, 268).

One potential limitation of Dsg3 CAAR-T-cell therapy in pemphigus IgG-driven autoimmunity not only against Dsg3 but also against Dsg1. Moreover, patients with Dsg3 mucosal dominant-PV were shown to possibly relapse as PF (or vice versa), probably via intermolecular epitope spreading (269, 270). These findings highlight the importance of targeting simultaneously both Dsg1 and Dsg3 reactive B-cells. A major challenge of CAR-T cell therapy in cancer is the ability of neoplastic cells to escape by down-regulating the expression of the target antigen. A novel strategy to overcome tumor antigen loss is modifying individual T cells with two distinct CAR molecules (dual-signaling CAR) or with one CAR molecule containing two different binding domains, referred to as Tandem CAR (271, 272). A similar strategy could be adopted to develop CAAR-T-cells targeting at the same time Dsg1 and Dsg3 reactive B-cells.

Also, another intriguing therapeutic perspective would be developing CAR-Treg cells to down-regulate ongoing immune reaction against Dsg (273, 274). In this regard, of interest is a study by Fransson et al. involving a murine model of autoimmune encephalitis, in which mouse CD4^+^ T cells were modified to express a CAR targeting myelin oligodendrocyte glycoprotein (MOG) in trans with the murine FoxP3 gene promoting Treg differentiation. Myelin targeting Treg cells successfully suppressed inflammation and improved symptoms in the treated mice (275).

Another potential limitation of CAAR-T-cell therapy in pemphigus includes prohibitive costs. For example, one single infusion of the CD19 CAR T-cell products costs around $ 373,000–475,000 (276). However, taking into account the economic impact of a virtually life-long immunosuppressive therapy, if the aim of pemphigus cure with front-line CAAR T-cell therapy were to be realized, reductions in cumulative spending may conceivably occur. Figure 9 represents how CAAR-T cells works in pemphigus.

### Targeting the Neonatal Fc Receptor in Pemphigus

FcRn is a heterodimer including the MHC class I-like H chain and the β_2_-microglobulin L chain (277). This receptor is involved in the transport of IgG from mother to fetus; different studies however have shown that FcRn also plays a critical role in regulating IgG homeostasis; accordingly, when IgG enter into FcRn-expressing cells, the IgG-FcRn binding in acidified endosomes avoids degradation of IgG and allows subsequent recycle and release of IgG in the extracellular space at a near-neutral pH. IgM and IgA do not bind to FcRn and consequently have a reduced half-life compared to IgG (278, 279). Blocking the interaction between FcRn and pathogenic autoantibodies in autoimmune diseases may thereby accelerate pathogenic autoantibody catabolism (280). Interestingly, keratinocytes have been shown to express FcRn (281). Indeed, different studies have demonstrated a critical role of FcRn in pemphigus. Knock-out mouse lacking FcRn do not develop acantholysis by passive transfer of anti-Dsg antibodies (282). Therapeutic efficacy of IVIg can be at least in part attributed to saturation of FcRn, allowing faster degradation of anti-Dsg autoantibodies (282). *In vitro*, an excess of normal IgG accordingly protects cultured keratinocytes from anti-Dsg autoantibodies-induced apoptolysis (283). One recent study demonstrated that FcRn induces enter of anti-mitochondrial antibodies, which are also found in pemphigus sera and contribute to keratinocyte shrinkage due to mithocondial damage. In this study, blocking of FcRn abolished the capacity of PV sera to cause detachment of keratinocytes *in vitro* (284). In another study, Recke et al. (285) found that an allelic variant harboring an amino acid replacement of His435 to Arg favoring high affinity of IgG3 to FcRn was associated with an increase risk of PV. Altogether these studies raise evidence for a potential benefit of targeting FcRn in pemphigus. In 2018, the results of a randomized double-blind placebo-controlled first in-human study on efgartigimod, an antagonist of FcRN, including 62 healthy volunteers showed that the drug was effective in reducing IgG levels by about 75% on after multiple dosing (286). The treatment was well-tolerated and no serious adverse events were recorded (286). A phase II study evaluating the safety and efficacy of ARGX-113, a human IgG1-derived Fc fragments binding to FcRn in patients with PV and PF is currently ongoing (NCT03334058) (287).

### Targeting T-Cells and the T-B Cell Cross Talk in Pemphigus

Autoreactive T-cells are critically involved in the pathogenesis of pemphigus (288). Both Th1 and Th2 cells reacting against Dsg were detected in patients with pemphigus at different stages of the disease. However, while Dsg3-reactive Th1 cells can be found in healthy individuals carrying PV predisposing HLA class II alleles, Dsg3-reactive Th2 cells are restricted to pemphigus patients (289, 290). In a recent study by our group, we demonstrated that patients with PV developed a predominant Th2-type response against Dsg3, in contrast to patients with lichen planus, who developed a Th1-type response against the identical autoantigen (291). Indeed, high amount of serum IL-4 sustaining autoantibody production by autoreactive B-cells have been found in pemphigus patients (292). Our group demonstrated the presence of IgE targeting Dsg3 in patients with pemphigus, which further supports the critical role of Th2 cells in orchestrating the inflammatory response and autoantibody production in pemphigus (293). Interestingly, dupilumab, a monoclonal antibody targeting IL-4 has been recently shown to be effective in bullous pemphigoid, a disease in which Th2 cells targeting BP180 and both IgG and IgE against BP180 have also a prominent pathogenic role (294, 295). As far as we are aware, no studies have yet investigated the efficacy of dupilumab in pemphigus. However, pathogenic evidence suggests that the drug may be of potential benefit (Figure 6). In parallel to activation of Th2 cells, in pemphigus there is a marked down-regulation of Dsg-specific Foxp3-expressing Treg cells (289). In mouse experiments, Treg cells created in Dsg3^−/−^ mice and transferred into mice with PV were able to reduce autoantibody production (296). Thus, enhancing Treg functions in pemphigus appears to be a promising strategy to restore the lost immune tolerance against Dsg. Infusion of autologous polyclonal regulatory T cells is currently being studied in a phase 1 open-label multicenter trial in patients with active pemphigus vulgaris and pemphigus foliaceus (NCT03239470) (135, 296).

Current work of our group aims at restoring immune tolerance to Dsg3 via targeting of autoreactive T cells. Specifically, we observed in a preclinical mouse model of pemphigus that injections of immunodominant HLA-DRß1^*^04:02-binding T cell epitopes of Dsg3 conjugated either to antigen-presenting cells lacking a second co-stimulatory signal or to nanoparticles prevented the production of pathogenic anti-Dsg3 IgG.

As previously mentioned, RTX has an inhibitory effect on the co-stimulation of autoreactive B and T-cells in pemphigus by depletion B cells which act as antigen presenting cells (140). In this process, the interaction between CD40 expressed on the surface of B-cells and CD40 L expressed on the surface of T-cells is thought to play an essential role (297). Interestingly, targeting CD40 L demonstrated efficacy in an active mouse PV model. Specifically, Rag2-deficient mice expressing Dsg3 and treated with a monoclonal antibody targeting CD40L did not develop PV after having received splenocytes from Dsg3 deficient mice. However, this preventive effect did not occur when the monoclonal antibody was given after the adoptive cell transfer suggesting that targeting T-B-cell cross-talk may be an effective tool to prevent disease recurrences in patients who have already achieved disease remission (Figure 9) (297–299).

### Is There a Possibility for a Local Targeted Therapy in Pemphigus?

Vinaj et al. (300) reported on 3 patients with oral PV lesions refractory to immunosuppressants, including intravenous RTX, in whom intralesional RTX injections led to meaningful clinical improvement, suggesting a local immunomodulating effect of the drug. A landmark paper by Yuan et al. (301) demonstrated an accumulation of Dsg-3 and Dsg-1 specific B-cells in the skin of pemphigus lesions. Additionally they showed that the skin in pemphigus serves as a tertiary lymphoid organ in which a close interaction between IL-21- and IL17A-producing CD4^+^ T cells leads to in loco production of anti-Dsg autoantibodies by auto-reactive B-cells. It is possible that the formation of this tertiary lymphoid organ in the skin may contribute to the resistance of pemphigus lesions to immunosuppressive treatments, including RTX. The discovery by Yuan et al. (301) may also provide a plausible explanation for the successful use of intralesional RTX in oral pemphigus lesions observed by Vinaj et al. (300). It is interesting to note that secretion of IL-21 is associated with activation of JAK1 and JAK3. Tofacinib, an inhibitor of both JAK1 and JAK3, was suggested as a possible therapeutic in pemphigus (302). Interestingly, tofacinib has been successfully applied in different autoimmune disorders (303, 304). Moreover, a topical preparation of tofacinib was shown to be effective in alopecia areata (305). Based on these findings, it would be intriguing to evaluate the potential benefit of topical JAK inhibitors as a local adjuvant therapy in pemphigus.

We have previously shown that mechanisms of acantholysis in pemphigus require activation of different intracellular signaling, including p38MAPK. *In vitro* studies showed that inhibition of p38MAPK eliminated PV IgG-induced blister formation in human skin biopsies (306). Oral p38MAPK inhibitors have been tried in clinical trials in rheumatoid arthritis, Crohn's disease and psoriasis, as well as in pemphigus, but had resulted in severe adverse events (1). Nevertheless, topical p38MAPK inhibitors have been shown to exert potent anti-inflammatory effects in the skin of mice following burn injury (307). Indeed, topical p38MAPK inhibitors may represent a therapeutic strategy in pemphigus to overcome the significant toxicity observed with the systemic counterpart. Finally in a study by Mao et al. (308), inhibition of STAT3 by hydrocortisone, rapamicin, an inhibitor of mTOR, or Stat3 inhibitor XVIII prevented blister formation in a passive transfer PV mouse model by up-regulating the expression of Dsg3. Interestingly, sirolimus, a systemic inhibitor of mTOR, was shown to be effective in PV, whereas topical rapamicin did not (309, 310). Notably, intratumoral injection of Stat3 oligonucletide decoy demonstrated mild efficacy in a trial including patients with head and neck tumors (311). Due to its pivotal implication in the mechanisms of acantholysis in pemphigus, Pharmacologic inhibition of Stat3 through topical drugs may also hold promise in the field of targeted therapy in pemphigus.

## Dedication

This article is dedicated to Stephen I. Katz, MD, PhD, who enormously contributed to a better understanding of autoimmune bullous skin diseases and immunological skin disorders in general. It was his institute which identified desmoglein 3 as the autoantigen of pemphigus vulgaris and firstly described the central role of autoreactive T cells in this disorder. Steve Katz trained world leaders in immunodermatology from all over the world and will be unforgotten as a superb mentor and wonderful person.

## Author Contributions

RM and MH designed the manuscript. DD, RM, RE, and MH drafted the manuscript and approved the final version of the manuscript. MH revised critically the final version of the manuscript.

### Conflict of Interest Statement

The authors declare that the research was conducted in the absence of any commercial or financial relationships that could be construed as a potential conflict of interest.
